# Motor recovery after activity-based training with spinal cord epidural stimulation in a chronic motor complete paraplegic

**DOI:** 10.1038/s41598-017-14003-w

**Published:** 2017-10-26

**Authors:** Enrico Rejc, Claudia A. Angeli, Darryn Atkinson, Susan J. Harkema

**Affiliations:** 1 Kentucky Spinal Cord Injury Research Center, University of Louisville, Louisville, Kentucky USA; 2 Department of Neurological Surgery, University of Louisville, Louisville, Kentucky USA; 3 Frazier Rehab Institute, Louisville, Kentucky USA

## Abstract

The prognosis for recovery of motor function in motor complete spinal cord injured (SCI) individuals is poor. Our research team has demonstrated that lumbosacral spinal cord epidural stimulation (scES) and activity-based training can progressively promote the recovery of volitional leg movements and standing in individuals with chronic clinically complete SCI. However, scES was required to perform these motor tasks. Herein, we show the progressive recovery of voluntary leg movement and standing without scES in an individual with chronic, motor complete SCI throughout 3.7 years of activity-based interventions utilizing scES configurations customized for the different motor tasks that were specifically trained (standing, stepping, volitional leg movement). In particular, this report details the ongoing neural adaptations that allowed a functional progression from no volitional muscle activation to a refined, task-specific activation pattern and movement generation during volitional attempts without scES. Similarly, we observed the re-emergence of muscle activation patterns sufficient for standing with independent knee and hip extension. These findings highlight the recovery potential of the human nervous system after chronic clinically motor complete SCI.

## Introduction

The prognosis for recovery of lower limb motor function in individuals with chronic clinically motor complete spinal cord injury (SCI) is poor^1,2^. Activity-based training (i.e. locomotor training with manual assistance provided by trainers) is not sufficient to promote significant restoration of motor function in this population^3–6^. However, it can induce positive neurophysiological adaptations in the spinal circuitry and, to some extent, modulate the motor pattern generated during assisted standing and stepping. Studies in animal models with complete SCI have demonstrated the potential of activity-based training to promote the recovery of lower limb motor function for standing and stepping^7–10^. This recovery is substantially improved with the combination of spinal cord epidural stimulation (scES) and also pharmacological intervention^11–15^. These findings led to the hypothesis that humans with motor complete SCI may be able to recover independent standing or stepping performing locomotor training with scES. We tested this hypothesis in four chronic clinically motor complete SCI individuals, and showed that stand training performed with individual-specific configurations optimized for standing (Stand-scES) progressively promoted the recovery of full body weight bearing standing with self-assistance for balance^16–18^. It was also observed that step training with scES optimized for stepping (Step-scES) performed subsequently to stand training impaired standing performance in three of these four participants, underscoring the relevance and task-specific effects of the trained motor task. In addition, delivering sub-motor threshold levels of scES customized for voluntary movement (Vol-scES) enabled these individuals to perform relatively fine volitional lower limb movements from the supine position in response to conceptual, auditory and visual input^16,19^. The presence of Stand-scES was always required to stand in all four individuals, and Vol-scES was always required to elicity voluntary leg movements in three of these research participants. This report describes the unexpected recovery of lower limb voluntary motor control and independent standing without scES in one of these chronic, motor complete SCI individuals that progressed throughout 44 months of activity-based training with scES.

## Materials and Methods

### Research participant

One of the four research participants with chronic motor complete SCI who were recruited to investigate the effects of activity-based training with scES on the recovery of lower limb motor function^18^ was enrolled to perform additional activity-based training with scES at home and in the laboratory following the completion of the initial study. Research participant B13, a 32-year old male, was implanted with a spinal cord epidural stimulation unit 4.2 years after SCI caused by a motorcycle accident. Prior to stimulator implant, this individual was unable to stand or walk independently or voluntarily move his legs despite standard-of-care rehabilitation and locomotor training received during the initial 21 months following the injury. Additional intensive locomotor training (80 sessions) performed prior to the stimulator implant did not result in functional improvements for standing, stepping or voluntary movement. Two clinicians independently performed a physical exam following the International Standards for Neurological Classification of Spinal Cord Injury^20,21^ prior to stimulator implant, and classified the individual as AIS B (pinprick and light-touch present below the lesion), with a neurological level of injury at C7. In addition, as reported in a previous publication^19^, no functional motor connectivity between the supraspinal and spinal centers below the level of injury was detected in this research participant. The individual signed an informed consent for electrode implantation, stimulation, activity-based training and physiological monitoring studies approved by the University of Louisville and the University of California, Los Angeles Institutional Review Boards. All research activities were performed in accordance with the guidelines and regulations of these Institutional Review Boards. The research participant and the other persons appearing in the Supplemental Videos included in this paper also gave written informed consent and granted full permission for their image to be used in publication online.

#### Surgical implantation of electrode array and stimulator

The lumbosacral enlargement was electrically stimulated during activity-based training using an epidural spinal cord stimulation unit (Restore ADVANCED, Medtronics) and a 16-electrode array (5-6-5 Specify, Medtronic) that was implanted at the T11-L1 vertebral level over the spinal cord segments L1-S1^16^. The electrode lead was tunneled to a subcutaneous abdominal pouch where the pulse generator was implanted. Representative scES parameters utilized during activity-based training are reported in Supplemental Table S1.

### Experimental Procedures

**Figure 1:**
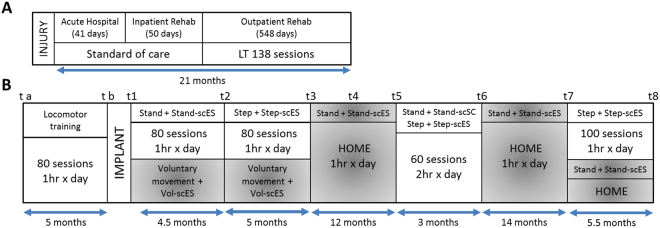
Experimental protocol timeline. Panel (A) Standard of care and outpatient rehabilitation (locomotor training, LT) within the initial 21 months since injury. Panel (B) Experimental sessions (t a to t8) and activity-based interventions performed prior to and after scES implant (see text for details).

The data reported in the present study were collected without scES at different time points, over a period of 4.1 years (Fig. 1). In particular, a series of experiments in the supine position were aimed at evaluating volitional motor control during attempts to perform unilateral (right) hip flexion and knee extension^22,23^. During hip flexion at experimental time points t1 to t5, a non-elastic cable was secured to the ankle and attached to a fixed frame; this cable allowed a maximum hip flexion of 30 degrees.

Another series of experiments were devoted to the assessment of lower limb muscle activation pattern generated during full weight-bearing standing and the amount of external assistance required for the individual to maintain upright position. Standing was performed overground using a custom designed standing apparatus comprised of horizontal bars anterior and lateral to the individual. These bars were used for upper extremity support and balance assistance. The participant initiated the sit to stand transition by positioning his feet shoulder width apart and shifting his weight forward to begin loading the legs. The participant used the horizontal bars of the standing apparatus during the transition phase to balance and to partially pull himself into a standing position. Trainers positioned at the pelvis and knees manually assisted as needed during the sit to stand transition. If the knees or hips flexed beyond the normal standing posture, external assistance at the knees distal to the patella was provided to promote knee extension, and at the hips below the iliac crest to promote hip extension and anterior tilt. Facilitation was provided either manually by a trainer or by elastic cords, which were attached between the two vertical bars of the standing apparatus.

#### Data acquisition

EMG, kinematics and ground reaction forces data were recorded at 2000 Hz using a custom-written acquisition software (National Instruments, Austin, TX). EMG activity of right (R) and left (L) gluteus maximus (GL), medial hamstring (MH), rectus femoris (RF), vastus lateralis (VL), tibialis anterior (TA), medial gastrocnemius (MG), soleus (SOL), sternocleidomastoid (SCM) and intercostal (sixth intercostal space; IC) muscles were recorded with bipolar surface electrodes with fixed inter- electrode distance^16^. Bilateral EMG from the iliopsoas (IL) was recorded with fine-wire electrodes. Lower limb joint angles were acquired using a high-speed optical motion capture system (Motion Analysis, Santa Rosa, CA). Ground reaction forces were collected using a high-resolution pressure sensing mat (HR mat system, TEKSCAN, Boston, MA) or two force platforms (Kistler Holding AG, Winterthur, Switzerland).

#### Data analysis

Data analysis of volitional attempts was performed on a 3-second time window, the onset of which coincided with the activation of the primary agonist muscle (iliopsoas for hip flexion attempts; vastus lateralis for knee extension attempts). If the primary agonist muscle was not active throughout the whole attempt, the time window onset corresponded to the beginning of the volitional attempt as estimated by the activation of intercostal or sternocleidomastoid muscles.

**Figure 2:**
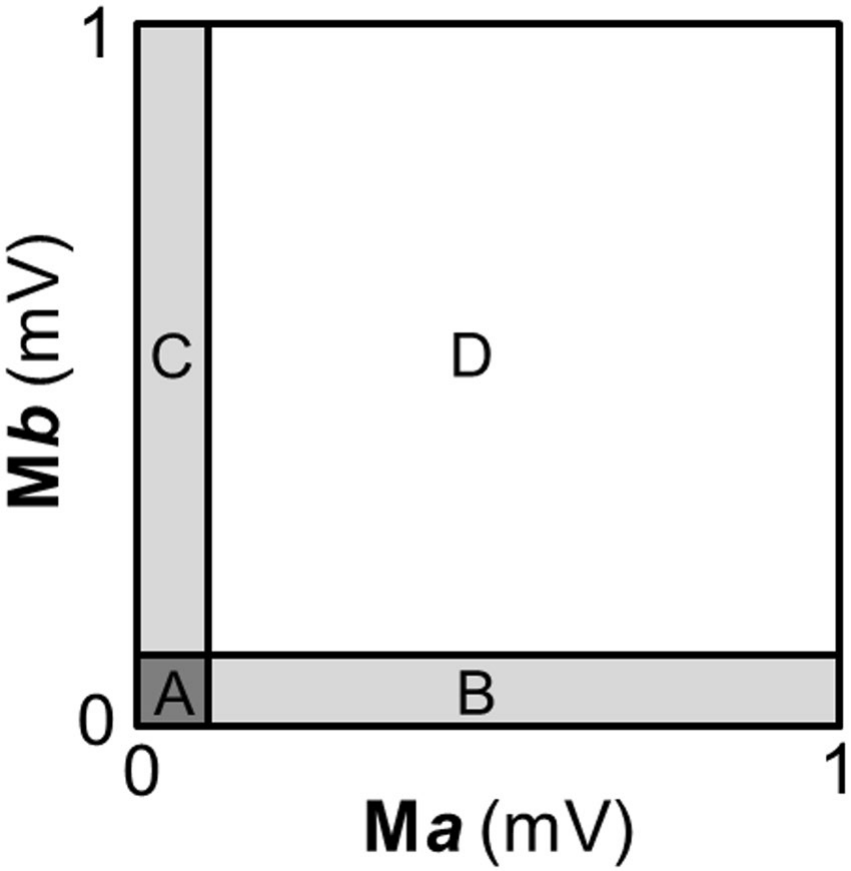
Quantitative joint probability density distributions analysis. The scatterplot obtained by the joint probability density distributions analysis (Hutchison *et al*.^24^), which describes the amplitude and temporal interrelationships of the EMG signals from two muscles (M*a* and M*b*), was divided into four areas (A,B,C and D) that were determined by setting a threshold equal to 10% of the of the EMG amplitude full scale values.

The amplitude of EMG activity during volitional attempts and standing was quantified by root mean square (RMS) and normalized (divided) by background (resting) RMS EMG amplitude that was recorded prior to the volitional attempts or during sitting, respectively. Hence, a normalized EMG amplitude value equal to 1 indicates that the EMG amplitude detected during the examined motor task (volitional movement attempt or standing) was equal to the background (resting) EMG amplitude. Joint probability density distributions (JPD) was calculated as reported by Hutchison and colleagues^24^ to obtain quantitative information about the coordination pattern between representative agonist and antagonist muscles during volitional movement attempts. Each data point in the JPD represents the amplitude relationship of the EMG signals from the two muscles at a given time point. Ten percent of the full scale value was then set as a threshold in order to define the four areas (A, B, C and D) represented in Fig. 2. The number of data points located in each of the four areas was finally expressed as a percentage of the total data points, representing the amount of isolated activation (area B and C) as well as the amount of co-contraction with low or high activation (area A and D, respectively) that occurred during a given attempt.

### Activity-based training

#### Locomotor training without scES in the laboratory

Prior to epidural stimulator implant (t a to t b, Fig. 1), the research participant underwent 80 sessions of locomotor training with body weight support (1 hour per session, five sessions per week), which included stand and step training (with stepping comprising the majority of time^25^). This training was intented to achieve any positive adaptations induced by activity-based rehabilitation before the beginning of training with scES.

#### Stand training with Stand-scES in the laboratory

After the stimulator implant, participant B13 underwent 80 sessions of full weight-bearing stand training (t1 to t2, Fig. 1). Stand training lasted 1 hour per session (5 sessions per week) and was always performed with Stand-scES using the custom designed standing frame described above. The individual was encouraged to stand for as long as possible throughout the training session, with the goal to stand for 60 minutes with the least amount of assistance. Seated resting periods occurred when requested by the individual. If, during standing, the participant’s knees or hips flexed beyond the normal standing posture, external assistance to facilitate hip and knee extension was provided either manually by a trainer or by elastic cords, which were attached between the two vertical bars of the standing frame.

#### Step training with Step-scES in the laboratory

Following the completion of stand training with Stand-scES, participant B13 performed 80 sessions of step training with body weight support (Innoventor, St. Louis, MO) on a treadmill (t2 to t3, Fig. 1). Step training (1 hour, 5 sessions per week) was always performed with Step-scES. The research participant stepped at body weight load and speed adapted to achieve appropriate stepping kinematics^25^. Stepping bout duration was dependent on participant’s endurance and stepping behavior. Following a stepping bout, participant B13 was encouraged to remain standing; body weight support and length of standing break varied. All trainers were careful to provide manual assistance only when needed following standard locomotor training principles^26^.

#### Voluntary movement training with Vol-scES

Voluntary lower extremity movement was practiced on a daily basis (about 1 hour per session, 5 sessions per week) in a home-based setting and once a week in the laboratory for 9.5 months (t1 to t3, Fig. 1). Unilateral leg flexion, ankle dorsiflexion and toe extension exercises were performed with task-specific scES configurations.

#### Stand training with Stand-scES at home

Following the completion of all laboratory sessions, the participant continued stand training with Stand-scES in the home base setting for 12 months (t3 to t5, Fig. 1). A custom built standing frame similar to the laboratory frame was provided to the research participant for standing. Elastic cords positioned on the standing frame provided assistance for hip and knee extension when needed. All stand training was performed with Stand-scES. On average, the research participant performed stand training at home for about 30 minutes per day. However, detailed information about the stand training distribution is not available.

#### Stand training and step training with scES in the laboratory

After 12 months of home-based training, the research participant returned to the laboratory for training (t5 to t6, Fig. 1). Stand training with Stand-scES and step training with Step-scES occurred daily (5 days per week, 1 hour per session) in an alternating fashion. Morning sessions were alternated between standing and stepping; the participant was then given a three-hour break before returning for the afternoon session, which was focused on the motor task not trained in the morning.

#### Stand training with Stand-scES at home

After the conclusion of the 3-month stand and step training in the laboratory, the research participant continued with home-based stand training with Stand-scES for the following 14 months (t6 to t7, Fig. 1). The home-based training setting was the same as that described for the first period of stand training at home (see above). The average duration of stand training was about 30 minutes per day.

#### Step training (in the laboratory) and stand training (at home) with scES

After 14 months of home-based stand training, the participant returned to the laboratory for additional step training with Step-scES (t7 to t8, Fig. 1). During this period, he also performed stand training with Stand-scES at home on a daily basis (5 days per week).

### Data Availability

All relevant data are within the paper and its Supporting Information files.

## Results

### Volitional movement attempts in the supine position

#### Hip flexion

**Figure 3:**
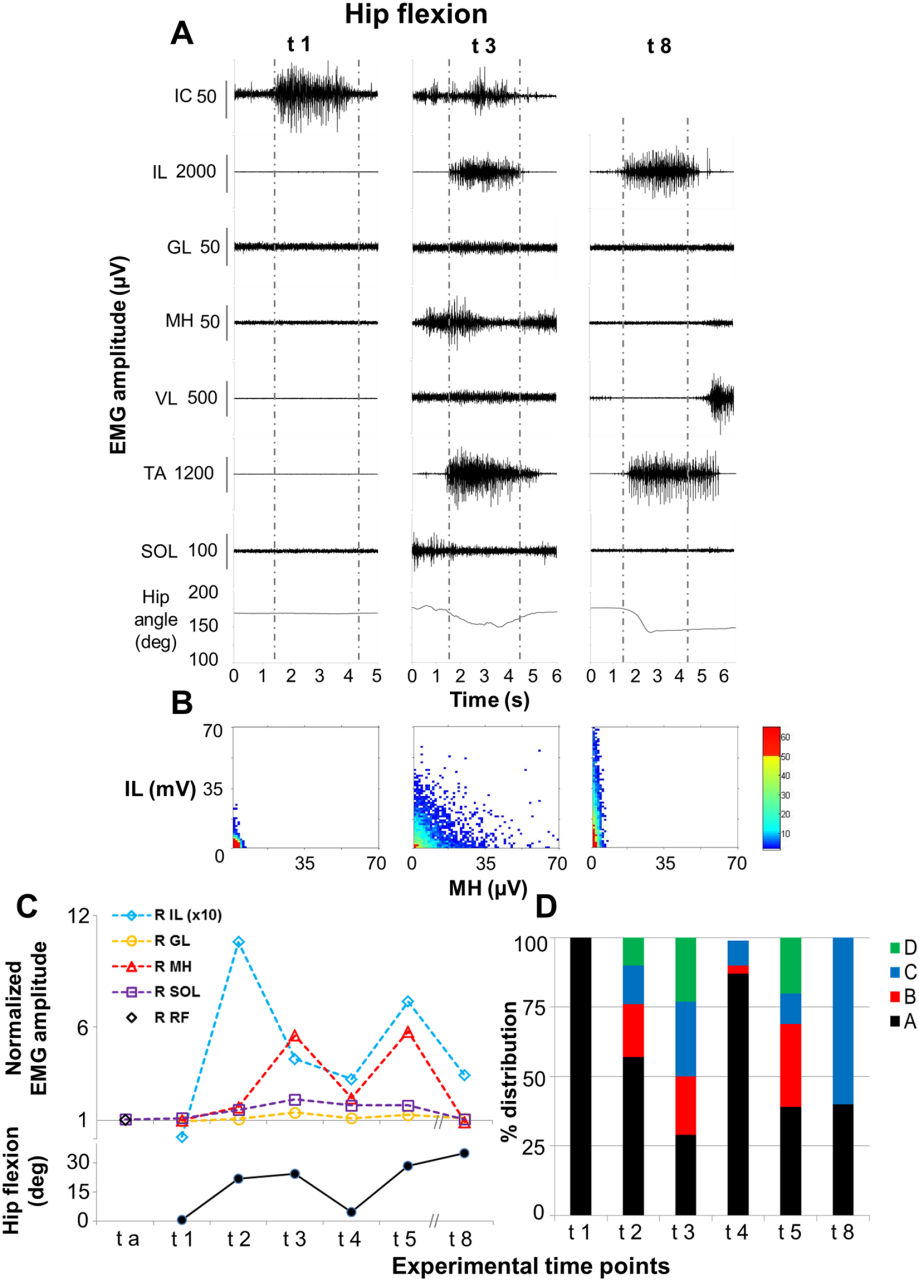
Volitional attempts to perform hip flexion. Panel (A) Electromyography (EMG) activity and hip joint angle recorded during representative attempts to volitionally perform right hip flexion in the supine position without scES after 80 sessions of locomotor training without scES (t1), after 9.5 months (t3) and after 44 months (t8) of activity-based training with scEs (see Fig. 1 for details). Panel (B) Probability density distribution of EMG amplitudes between the iliopsoas (IL, hip flexor) and the medial hamstrings (MH, hip extensors) calculated during the volitional attempts (data comprised between the two vertical grey dotted lines in Panel A) performed at the experimental time points t1, t3 and t8. Panel (C) EMG amplitude recorded during the volitional attempts, normalized by background (resting) EMG amplitude, and the resulting amount of hip flexion. Kinematics was not recorded at experimental time point t a. Panel (D) Quantitative probability density distribution of EMG amplitudes between iliopsoas and medial hamstrings calculated during the volitional attempts. Black and green indicate the amount of co-contraction at low or high level of activation, respectively (area A and D showed in Fig. 2). Blue and red indicate the isolate activation of iliopsoas or medial hamstrings, respectively (area C and B showed in Fig. 2). EMG was recorded from the following muscles of the right lower limb: IL, iliopsoas; GL, gluteus maximus; MH, medial hamstring; VL, vastus lateralis; TA, tibialis anterior; SOL, soleus. At the experimental time point t a, rectus femoris (RF) was monitored instead of IL as representative hip flexor. EMG activity from intercostal (IC) muscle was recorded to monitor the volitional effort onset.

The intent to execute a hip flexion movement in the supine position did not induce any modulation of EMG activity recorded from the lower limb muscles before and after locomotor training performed without scES (ta and t1, Fig. 3A and C). Conversely, after stand training with Stand-scES and also after step training with Step-scES (t2 and t3, respectively), the volitional hip flexion attempts led to the co-activation of proximal muscles, including the primary hip flexor (IL) and its antagonist MH (Fig. 3A–C), as well as distal muscles (i.e. TA). This activation pattern resulted in the actual flexion of the lower limbs (Supplemental video S2). The EMG pattern recorded at t3 (Fig. 3A) was similar to that observed at t2, with higher EMG amplitude in MH, lower EMG amplitude in IL, and decreased amount of co-contraction at low level of activation between MH and IL (“A” in Fig. 3D) at t3. Lower EMG amplitude was recorded from IL and MH during the attempt to flex the hip joint at t4 (Fig. 3C); this activation pattern coincided with the inability to generate an actual hip flexion movement. Conversely, the motor pattern observed at t5 was more similar to that recorded at t3 in terms of amount of hip flexion (28 vs 24 deg), EMG amplitude and coordination between IL and MH (Fig. 3C and D). Interestingly, the EMG pattern observed at t8 differed substantially from the previous ones, as the antagonist muscle MH and some distal muscles (i.e. SOL, but not TA) showed lower EMG activity during hip flexion (Fig. 3C). Also, the coordination pattern was different, describing a predominant activation of IL without the simultaneous activation of MH (Fig. 3B and D).

#### Knee extension

**Figure 4:**
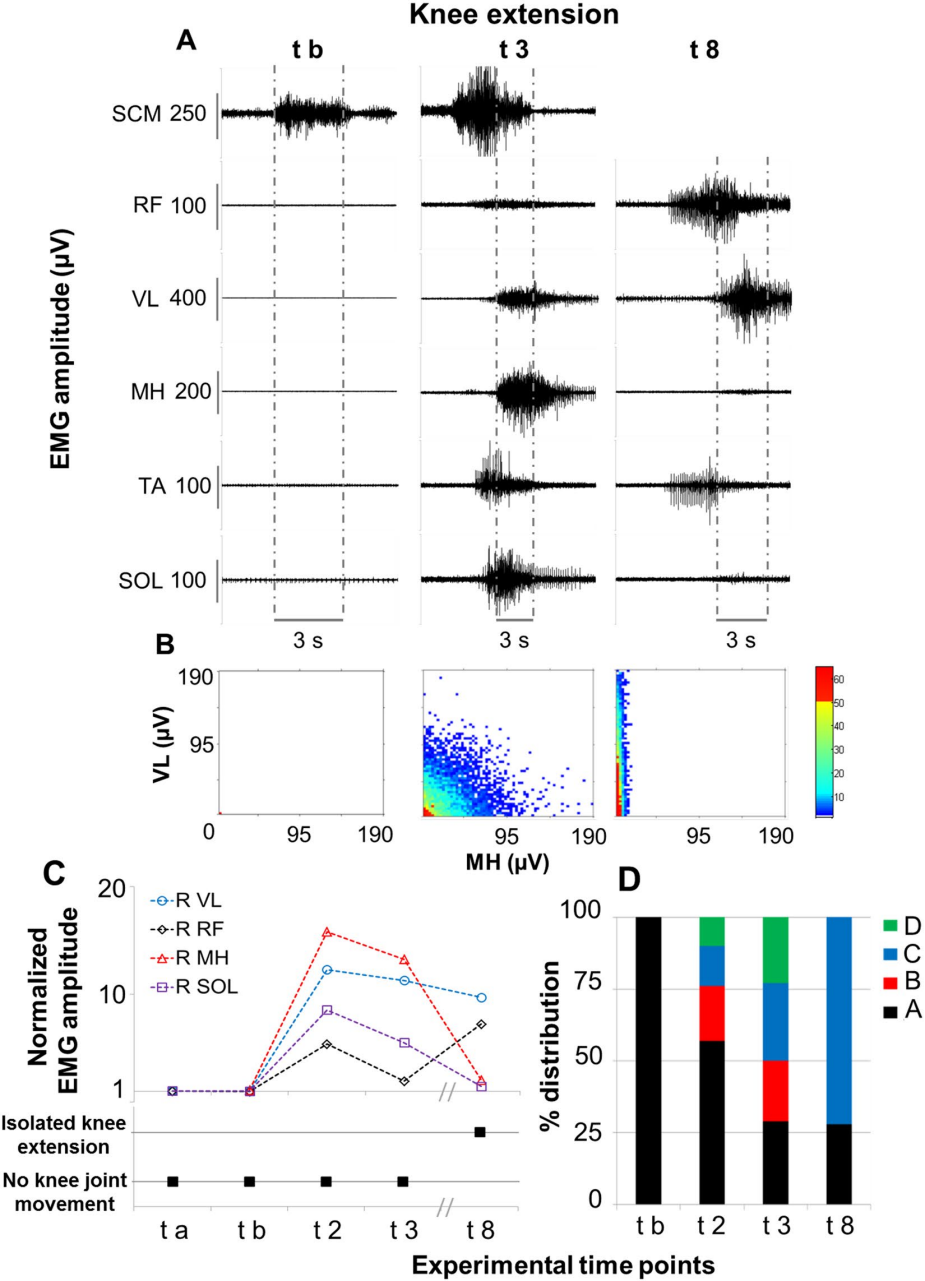
Volitional attempts to perform knee extension. Panel (A) Electromyography (EMG) activity recorded during representative attempts to volitionally perform right knee extension in the supine position without scES after 80 sessions of locomotor training without scES (t b), after 9.5 months (t3) and after 44 months (t8) of activity-based training with scEs (see Fig. 1 for details). Panel (B) Probability density distribution of EMG amplitudes between the vastus lateralis (VL, knee extensor) and the medial hamstrings (MH, knee flexors) calculated during the volitional attempts (data comprised between the two vertical grey dotted lines in Panel A) performed at the experimental time points t b, t3 and t8. Panel (C) EMG amplitude recorded during the volitional attempts, normalized by background (resting) EMG amplitude, and the resulting knee joint movement. Panel (D) Quantitative probability density distribution of EMG amplitudes between the vastus lateralis and the medial hamstrings calculated during the volitional attempts. Black and green indicate the amount of co-contraction at low or high level of activation, respectively (area A and D showed in Fig. 2). Blue and red indicate the isolate activation of vastus lateralis or medial hamstrings, respectively (area C and B showed in Fig. 2). EMG was recorded from the following muscles of the right lower limb: RF, rectus femoris; VL, vastus lateralis; MH, medial hamstring; TA, tibialis anterior; SOL, soleus. EMG activity from sternocleidomastoid (SCM) muscle was recorded to monitor the volitional effort onset.

Volitional attempts to perform knee exension in the supine position were also investigated at different experimental time points (Fig. 4). The intent to execute this motor task did not induce any modulation of EMG activity recorded from the lower limbs before and after locomotor training performed without scES (ta and tb, Fig. 4A and C). Conversely, after stand training with Stand-scES (t2), the research participant showed the ability to co-activate different lower limb muscles when attempting to extend the knee joint, including the main agonist (VL) and antagonist (MH) muscles (Fig. 4B and C); however, no actual joint movement resulted from this activation pattern. As observed during hip flexion attempts, the motor pattern recorded at t2 and t3 was similar, with decreased amount of co-contraction at low level of activation between VL and MH (“A” in Fig. 4D) observed at t3. Another similarity between hip flexion and knee extension attempts can be observed at t8, when knee extensors (VL and RF) showed relevant EMG amplitude during the volitional attempt, while MH as well as other distant muscles (i.e. SOL) presented negligible EMG activity. The coordination pattern between VL and MH was also different from the previous time points, with a predominant and isolated activation of VL without the concurrent activation of MH (Fig. 4B and D). This activation pattern coincided with the actual extension of the knee joint (Supplemental Video S2).

### EMG activity and external assistance during standing

**Figure 5:**
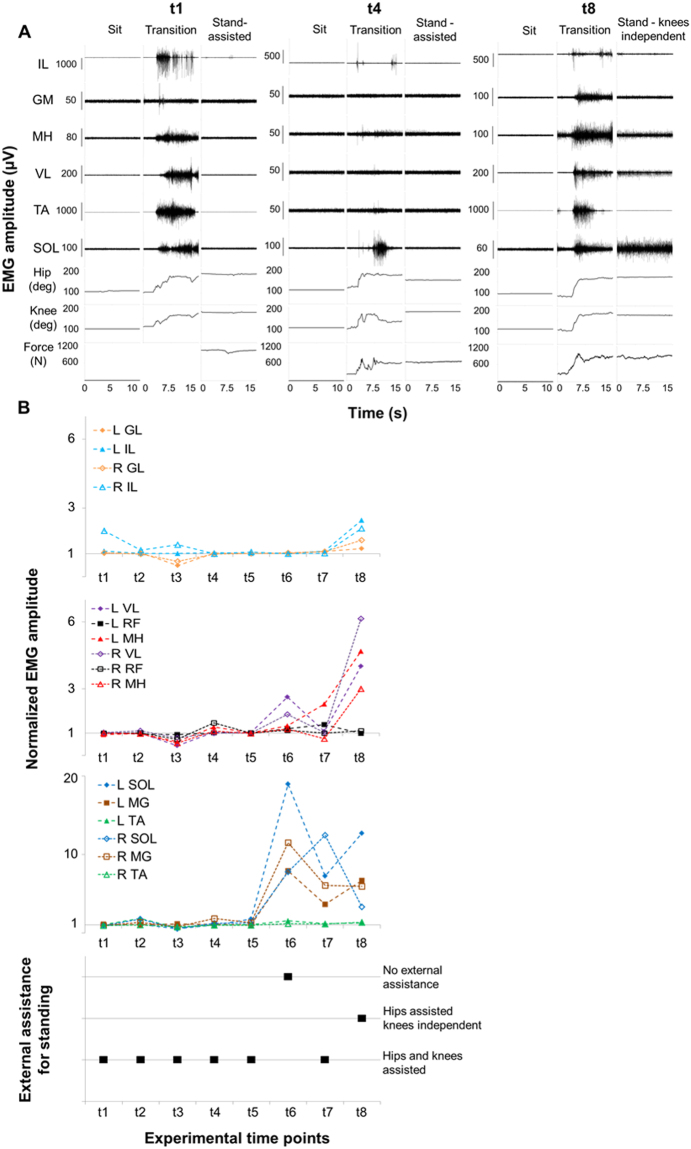
Time course of EMG amplitude and external assistance during standing. Panel (A) Electromyography (EMG) activity, hip and knee joint angle, and ground reaction forces recorded during sitting, sit-to-stand transition and overground full weight-bearing standing without epidural stimulation prior to any training (t1), after 15.5 months (t4) and after 44 months (t8) of activity-based training with scEs (see Fig. 1 for details). Panel (B) Time course of EMG amplitude recorded during standing, normalized by background (resting) EMG amplitude, and amount of external assistance needed for standing without epidural stimulation. EMG was recorded from the following muscles of the left (L) and right (R) lower limb: IL, iliopsoas; GL, gluteus maximus; MH, medial hamstring; VL, vastus lateralis; RF: rectus femoris; TA, tibialis anterior; SOL, soleus; MG: medial gastrocnemius.

**Figure 6:**
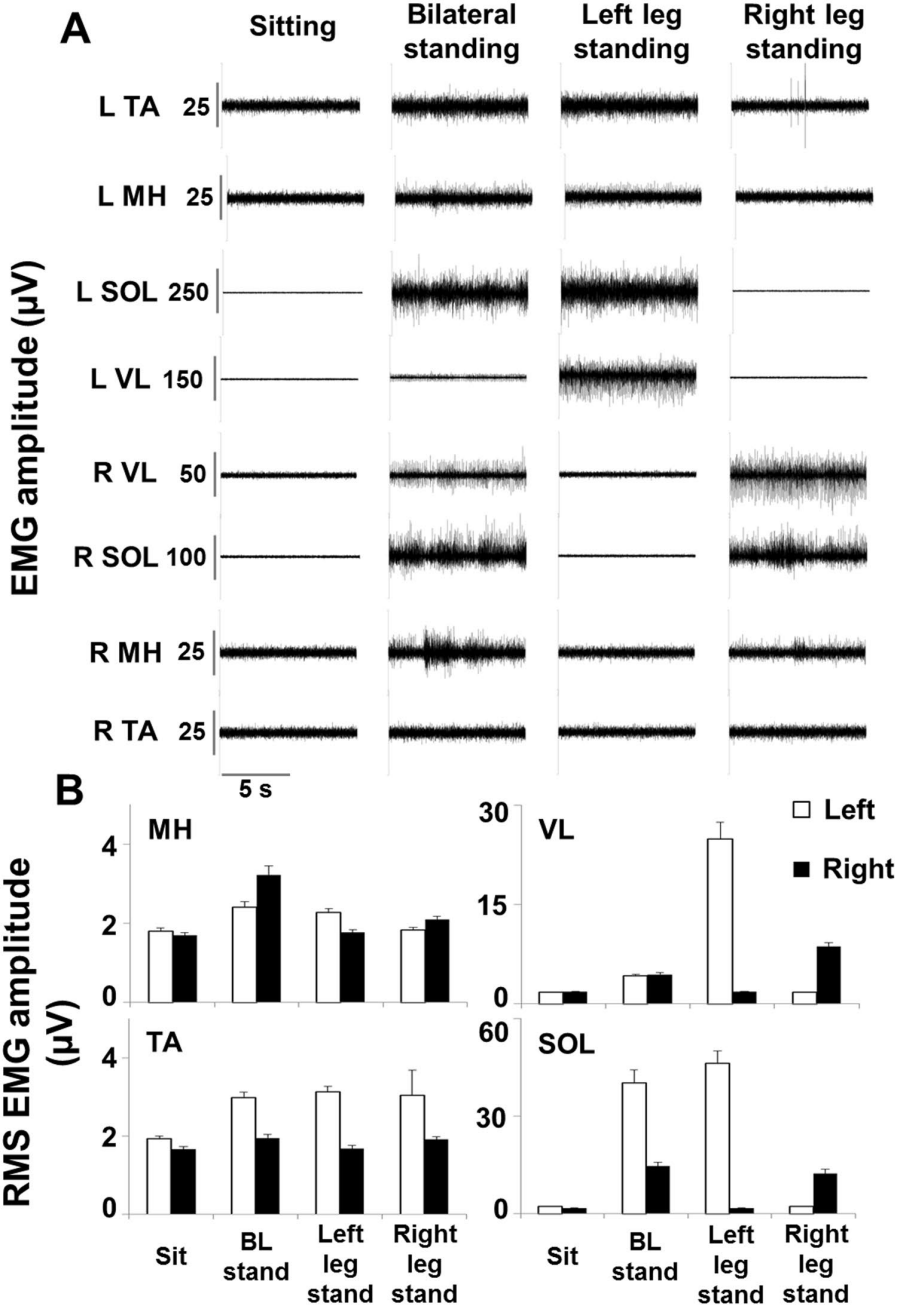
EMG activity during bilateral and unilateral independent standing. Panel (A) Electromyography (EMG) activity recorded during sitting, bilateral and unilateral full weight-bearing independent standing without epidural stimulation after 29.5 months of activity-based training with scEs (t6, see Fig. 1 for details). Panel (B) EMG amplitude calculated as the root mean square (RMS) over 10 seconds of steady sitting, bilateral and unilateral independent standing. EMG was recorded from the left (L) and right (R) medial hamstring (MH), vastus lateralis (VL), tibialis anterior (TA) and soleus (SOL).

When the research participant performed a sit to stand transition, lower limb EMG activity was usually observed during the transition phase at every time point (Fig. 5A). On the other hand, little or no EMG activity was detected during both sitting and assisted standing at the experimental time points t1 to t5, as exemplified in Fig. 5A and quantified in Fig. 5B. In particular, the upright position was achieved and maintaind via external assistance for hip and knee extension provided by trainers and/or elastic cords (Supplemental Video S3). Interestingly, at t6, continuous EMG activity was recorded from different lower limb muscles of the ankle and knee joint when the standing position was achieved (Figs 5B and 6). This change in EMG pattern coincided with the participant’s ability to stand bearing full body weight without any external assistance, placing his upper limbs on the standing frame for balance control (Supplemental Video S3). A similar muscle activation pattern, without ability to stand independently, was recorded at t7, while greater EMG activity of thigh muscles and independence of knee extension was detected at t8 (Fig. 5A and B).

At experimental time point t6, we also tested the participant’s ability to stand unilaterally. Full body weight was supported by one leg without external assistance while the knee of the contralateral leg was bent through external assistance (Supplemental Video S3). Interestingly, the primary antigravity muscles (SOL and VL) of the loaded leg showed EMG amplitudes that tended to be greater than those recorded during bilateral standing, while negligible EMG activity (similar to that recorded in sitting) was observed in the same muscles of the unloaded leg (Fig. 6A and B). On the other hand, representative flexor muscles (MH and TA) showed a limited increase in EMG amplitude during standing as compared to sitting, as well as a limited EMG modulation due to the leg loading or unloading while standing unilaterally.

## Discussion

We observed substantial recovery of volitional lower limb motor control and independent standing ability without using scES in an individual with chronic, motor complete SCI thoughout long-term activity-based training with task-specific scES parameters. At the beginning of the study, attempts to perform hip flexion or knee extension without scES did not induce any volitional movement or EMG activity from the lower limb muscles (Figs 3 and 4). However, participant B13 was able to execute volitional lower limb movements in presence of Vol-scES after only 4 days of testing^19^. Similar outcomes were observed in two additional chronic, motor complete individuals. These findings suggest that even at chronic timepoints, anatomical connections across the lesion may persist in individuals with motor complete (AIS B), or motor and sensory complete (AIS A) lesions^23,27–29^. However, these descending inputs were not sufficient to activate motor pools in the absence of scES.

Interestingly, stand training with Stand-scES and voluntary movement training with Vol-scES were sufficient to modify the motor output generated during volitional attempts to move the lower limbs without spinal stimulation. In fact, the intent to perform hip flexion and knee extension at experimental time point t2 resulted in the co-activation of agonist and antagonist muscles as well as distant leg muscles, and in the ability to perform hip flexion from the supine position (Figs 3 and 4). Similar motor patterns were observed until the experimental time point t5. The re-emergence of volitional leg muscle activation appears promoted by activity-based training with scES; different mechanisms may have been involved in this adaptation, including strengthening of residual supraspinal influence via plasticity in local spinal circuits, and/or plasticity in descending pathways (i.e. sprouting and/or growth of axons from supraspinal centers to interneuronal and/or motorneuronal pools located below the lesion)^15,30–33^. Co-activation of agonist, antagonist and distant muscles as well as multi-joint movements of the lower limb are also often observed in clinically motor incomplete SCI individuals when attempting to perform single-joint movements^34,35^. The inability of these individuals to perform isolated muscle activation leading to single-joint movements may suggest that remaining supraspinal motor input to the spinal cord was sufficient to initiate a motor pattern, but had limited capacity to selectively activate specific muscles of a single joint or to simultaneously inhibit antagonist muscles. At t8, further improvements in the volitional motor patterns were observed. In particular, during both hip flexion and knee extension attempts, a substantial decrease in the co-activation of antagonists and distant muscles was detected (Figs 3 and 4), along with increased EMG amplitude of prime movers (RF and VL) and actual joint movement during knee extension attempts. Long-term activity-based training with scES may have been promoted a more selective activation pattern via remodeling of synaptic connections among spinal inhibitory and excitatory interneurons projecting to motorneurons^36^. In addition, other mechanisms of inhibitory control that are typically impacted by SCI (i.e. nonreciprocal Ib inhibition^37^; reciprocal or ‘disynaptic’ inhibition^38^) also may have been reorganized by long-term activity-based training with scES, having a role in the observed refinement of volitional control^39^.

Limited recovery of volitional motor control after chronic motor complete SCI were observed in one individual after a 3-year period of functional electrical stimulation bicycle therapy^40^, in 5 individuals after activity-based training with transcutaneous spinal cord electrical stimulation combined with pharmacological intervention^36^, in three individuals after functional electrical stimulation-assisted locomotor training and continuous pelvic-lumbosacral neuromodulation^41^, and in a sub-group of individuals after long-term activity-based interventions enriched by virtual reality and multi-sensory feedback^42^. All these interventions activated the neuromuscular system below the level of injury with activity-based training. The time course of assessments presented in this study uniquely demonstrates the re-emergence of volitional motor control and its evolution over time, which seem to mirror the pattern of recovery frequently observed after incomplete SCI^39^. These findings suggest that when there is no ability to voluntarily generate movement below the level of injury, residual descending input to the spinal circuitry may be accessed with intense activity-based interventions to result in plasticity that can promote volitional movement recovery.

The loss of standing ability is also a dramatic consequence after severe SCI in humans. Chronic motor complete SCI individuals do not recover overground standing or walking, even after intense activity-based training (i.e. locomotor training)^3–6^. Similar findings were observed in the present study at experimental time points t1 to t5. In fact, the research participant was able to achieve and maintain the upright position only via external assistance for hips and knees extension. In addition, EMG activity recorded during assisted standing at the experimental time points t1 to t5 was negligible and comparable to that recorded in sitting (Fig. 5). Surprisingly, relevant motor pattern adaptations were observed during standing at t6 (Figs 5 and 6). In particular, continuous EMG activity was recorded from primary antigravity muscles (i.e. ankle plantarflexors and VL), which resulted in the individual’s ability to maintain hip and knee extension independently while standing, placing his upper limbs on the standing frame for balance control (Supplemental Video S3). Similar muscle activation patterns were also observed at t7 and t8. While the mechanisms underlying this motor recovery are currently unclear, a recent review by Cote and colleagues^43^ supports the view that humans are particularly dependent on residual descending input to generate stepping and standing, and that the human lumbosacral spinal circuitry is more depressed after a severe SCI, compared to other mammals. This assumption is also empirically supported by the fact that tonic, near-motor threshold lumbosacral scES aimed at mimicking the supraspinal tonic drive to the lumbosacral spinal circuitry enabled the generation of motor patterns effective for standing in individuals with chronic, motor complete SCI^17,18^. Propriospinal neurons may have played a role in the observed motor function recovery^43^, contributing to the formation of novel relays by receiving input from damaged descending axons and transmitting the information to the caudal cord in response to long-term activity-based training with scES^30,44,45^. In spite of the different mechanisms possibly involved in the training-induced improvement of functional motor connectivity between the supraspinal and spinal centers below the level of injury, it can be hypothesized that this improved connectivity, combined with weight bearing-related sensory information, may have promoted a level of lumbosacral spinal network excitability sufficient for the generation of motor pattern effective for standing.

Activity-based training is one of the main determinants of the motor improvements observed in the present study. However, little is known about the influence of different components of activity-based training (i.e. volume, intensity, frequency) on physiological adaptations resulting in motor function improvements after SCI^43^. In the present study, one of the most relevant improvements, the generation of motor patterns sufficient for standing independently, was observed for the first time after a 3-month training period (t5 to t6) that began 21.5 months after stimulation implant. It is worth noting that this training paradigm differed substantially from previous training in terms of: *i)* frequency of the training stimulus; *ii)* motor tasks trained in a day; and *iii)* volitional involvement of the research participant. In particular, during this period, two 1-hour training sessions per day were performed (1 stand training with Stand-scES and 1 step training with Step-scES) instead of the 1-hour daily training previously performed. The choice to train daily both standing and stepping rather than focusing on one motor task was based on previous findings that showed as standing ability impaired after a period of step training in three out of four motor complete SCI individuals implanted with a scES unit^18^. Hence, we proposed to train both standing and stepping in an alternating fashion with the hypothesis that this approach would promote improvements in both motor tasks. In addition, the research participant was strongly encouraged to volitionally contribute to the modulation of activation pattern during standing and stepping. In fact, it was previously shown that EMG pattern during assisted stepping with scES can be dramatically modulated by the intent of the clinically motor complete individual to contribute to the stepping pattern generation^19^, although this approach could lead to an overall impaired motor output (i.e. greater co-contraction during assisted stepping). Another difference was that the main goal of this training paradigm was no longer to complete the training session with the least amount of seated rest regardless of independence level, but rather to decrease external assistance regardless of the amount of seated rest required. Motivation and goal-oriented training were shown as important for the recovery of voluntary control of locomotion after SCI in rats, inducing growth of *denovo* brainstem and intraspinal relays^15^. Conversely, rats that were trained in a more passive and automated manner (treadmill-restricted training), which did not promote oriented cortical activity, did not show functional recovery overground or neural plasticity across the lesion. While in the present study we have not collected any data related to cortical activity, we have observed that the research participant attention during training sessions performed between t5 and t6, and between t7 and t8, was markedly focused on the trained motor task, attempting to volitionally contribute to the motor output. Therefore, it can be hypothesized that the increased volitional involvement of the research participant during stand and step training may have also contributed to the improved motor execution observed between t6 and t8, possibly promoting further intra- and supra-spinal plasticity.

In conclusion, long-term activity-based training with scES promoted recovery of both volitional lower limb movements and standing without using scES in an individual with a chronic, motor complete SCI. In particular, this is the first report detailing the ongoing neural adaptations that allowed a functional progression from no volitional muscle activation to a refined, task-specific activation pattern during volitional attempts, as well as to the generation of activation patterns sufficient for standing. Novel characteristics of the training paradigm that were implemented after experimental time point t5 (i.e. greater volitional involvement of the participant; greater training frequency) were conceivably important for the functional improvements herein described. These findings highlight the remarkable recovery potential of the human nervous system after chronic clinically motor complete SCI in response to activity-based training with scES, and have clear implications for the rehabilitation community. Future studies should attempt to better understand the influence of different activity-based training components (i.e. volume, intensity, frequency) on the physiological adaptations resulting in motor function improvements after severe SCI.

## Electronic supplementary material

Supplementary Information

Supplemental video S2

Supplemental video S3
