# Supplementary material for: Motor recovery after activity-based training with spinal cord epidural stimulation in a chronic motor complete paraplegic
Source: Sci Rep. 2017 Oct 26;7:13476. doi: 10.1038/s41598-017-14003-w (PMC5658385; doi:10.1038/s41598-017-14003-w)
Supplement: Supplementary file 1 — Supplementary Information [file 41598_2017_14003_MOESM1_ESM.pdf]

# Title

Motor recovery after activity-based training with spinal cord epidural stimulation in a chronic motor complete paraplegic.

Enrico Rejc[1,2, #], Claudia A. Angeli[3,1, #], Darryn Atkinson[1,2], Susan J. Harkema[1,2,3 *]

[1] Kentucky Spinal Cord Injury Research Center, University of Louisville, Louisville, Kentucky, USA.

[2] Department of Neurological Surgery, University of Louisville, Louisville, Kentucky, USA.

[3] Frazier Rehab Institute, Louisville, Kentucky, USA.

*# Equal contribution*

** Corresponding Author*

## Supplementary Information

**Supplemental table S1:** Representative spinal cord epidural stimulation parameters utilized during stand training (Stand-scES), step training (Step-scES) and voluntary movement training (Vol-scES) throughout 3.7 years of activity-based interventions (t1 to t8, see text for details). Electrode configuration (cathodes in black, anodes in red, inactive in white), stimulation frequency and intensity are reported. From experimental time point t5 onward, Stand-scES and Step-scES included multiple programs (P1 to P4) that were delivered sequentially by the same electrode array. Task-specific epidural stimulation parameters are not reported ( // ) for the time periods during which a motor task was not trained.

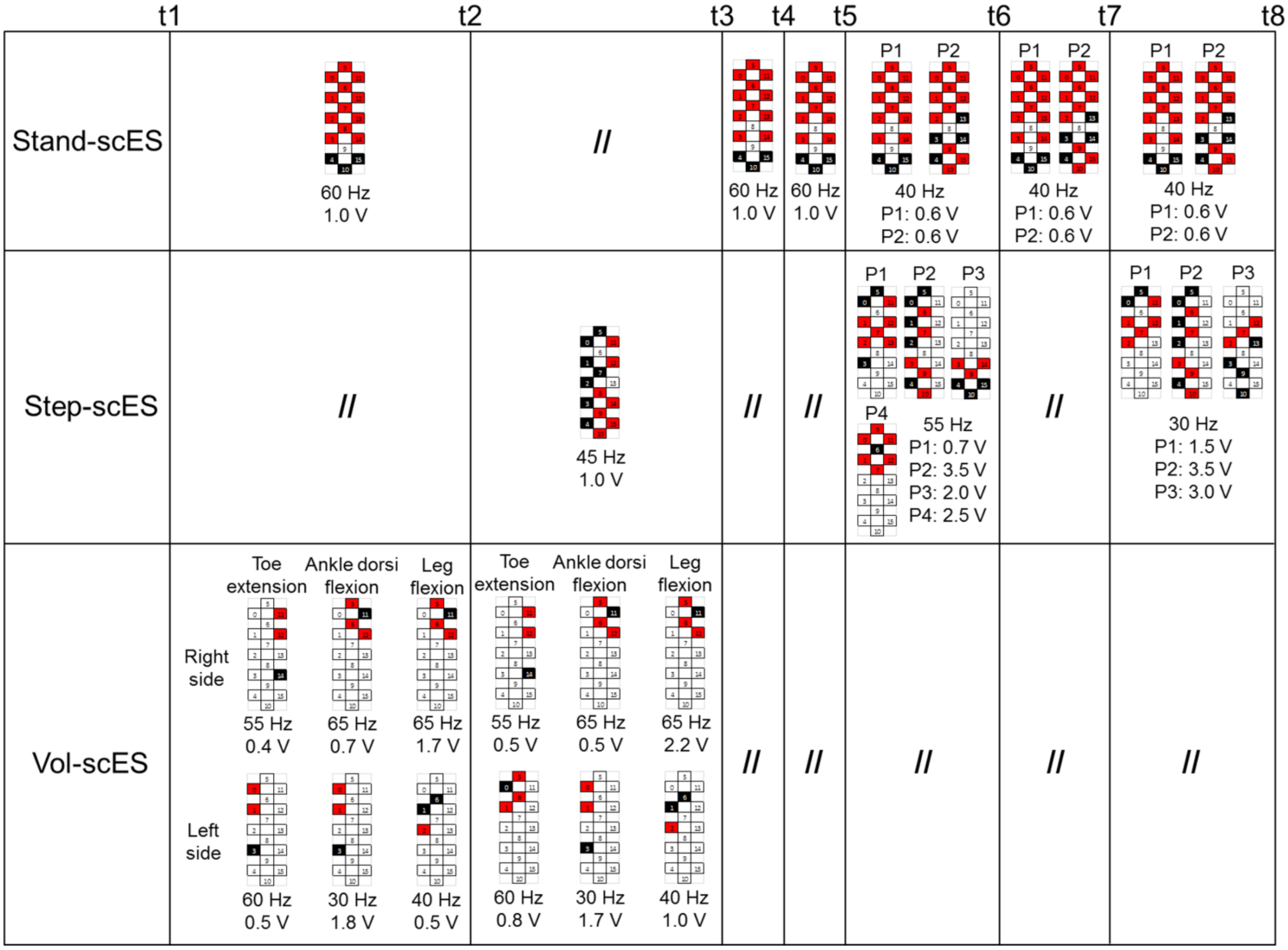

| | t1–t2 | t2–t3 | t3–t4 | t4–t5 | t5–t6 | t6–t7 | t7–t8 |
|---|---|---|---|---|---|---|---|
| Stand-scES | 60 Hz<br>1.0 V | // | 60 Hz<br>1.0 V | 60 Hz<br>1.0 V | P1 P2<br>40 Hz<br>P1: 0.6 V<br>P2: 0.6 V | P1 P2<br>40 Hz<br>P1: 0.6 V<br>P2: 0.6 V | P1 P2<br>40 Hz<br>P1: 0.6 V<br>P2: 0.6 V |
| Step-scES | // | 45 Hz<br>1.0 V | // | // | P1 P2 P3 P4<br>55 Hz<br>P1: 0.7 V<br>P2: 3.5 V<br>P3: 2.0 V<br>P4: 2.5 V | // | P1 P2 P3<br>30 Hz<br>P1: 1.5 V<br>P2: 3.5 V<br>P3: 3.0 V |
| Vol-scES | Right side: Toe extension 55 Hz 0.4 V; Ankle dorsi flexion 65 Hz 0.7 V; Leg flexion 65 Hz 1.7 V<br>Left side: 60 Hz 0.5 V; 30 Hz 1.8 V; 40 Hz 0.5 V | Toe extension 55 Hz 0.5 V; Ankle dorsi flexion 65 Hz 0.5 V; Leg flexion 65 Hz 2.2 V<br>60 Hz 0.8 V; 30 Hz 1.7 V; 40 Hz 1.0 V | // | // | // | // | // |

**Supplemental video S2:** Volitional attempts to perform right hip flexion at experimental time points t2 and t8; volitional right knee extension at t8.

**Supplemental video S3:** Sit to stand transition and standing with external assistance for hip and knee extension at t5; sit to stand transition and standing with independent hip and knee extension at t6; single leg stance at t6.
